# Towards development of treat to target (T2T) in childhood-onset systemic lupus erythematosus: PReS-endorsed overarching principles and points-to-consider from an international task force

**DOI:** 10.1136/ard-2022-223328

**Published:** 2023-01-10

**Authors:** Eve Mary Dorothy Smith, Amita Aggarwal, Jenny Ainsworth, Eslam Al-Abadi, Tadej Avcin, Lynette Bortey, Jon Burnham, Coziana Ciurtin, Christian M Hedrich, Sylvia Kamphuis, Deborah M Levy, Laura B Lewandowski, Naomi Maxwell, Eric F Morand, Seza Ozen, Clare E Pain, Angelo Ravelli, Claudia Saad Magalhaes, Clarissa A Pilkington, Dieneke Schonenberg-Meinema, Christiaan Scott, Kjell Tullus, Michael William Beresford, the cSLE T2T International Task Force, Beatrice Goilav

**Affiliations:** 1 Department of Women’s & Children’s Health, Institute of Life Course and Medical Sciences, University of Liverpool Faculty of Health and Life Sciences, Liverpool, UK; 2 Department of Paediatric Rheumatology, Alder Hey Children's NHS Foundation Trust, Liverpool, UK; 3 Department of Clinical Immunology & Rheumatology, Sanjay Gandhi Post Graduate Institute of Medical Sciences, Lucknow, Uttar Pradesh, India; 4 Department of Paediatric Rheumatology, Birmingham Children's Hospital NHS Foundation Trust, Birmingham, UK; 5 Department of Pediatric Rheumatology and Clinical Immunology, University Medical Centre Ljubljana Division of Paediatrics, Ljubljana, Slovenia; 6 TARGET Lupus Public Patient Involvement and Engagement Group, University of Liverpool Faculty of Health and Life Sciences, Liverpool, UK; 7 Department of Pediatric Rheumatology, The Children's Hospital of Philadelphia, Philadelphia, Pennsylvania, USA; 8 Centre for Adolescent Rheumatology Versus Arthritis, Division of Medicine, University College London, London, UK; 9 Department of Paediatric Rheumatology, Erasmus Medical Center, Rotterdam, The Netherlands; 10 Department of Pediatric Rheumatology, The Hospital for Sick Children, Toronto, Ontario, Canada; 11 Department of Pediatric Rheumatology, University of Toronto, Toronto, Ontario, Canada; 12 National Institutes of Health, National Institute of Arthritis and Musculoskeletal and Skin Diseases Systemic Autoimmunity Branch, Bethesda, Maryland, USA; 13 Department of Rheumatology, Monash University, Clayton, Victoria, Australia; 14 Department of Pediatrics, Division of Rheumatology, Hacettepe University Faculty of Medicine, Ankara, Turkey; 15 Direzione Scientifica, Istituto Giannina Gaslini Istituto Pediatrico di Ricovero e Cura a Carattere Scientifico, Genova, Liguria, Italy; 16 Dipartimento di Neuroscienze, Riabilitazione, Oftalmologia, Genetica e Scienze Materno Infantili (DINIGMI), Università degli Studi di Genova, Genova, Italy; 17 Department of Pediatric Rheumatology, Botucatu Medical School, Sao Paulo University Faculty of Medicine, Sao Paulo, Brazil; 18 Department of Paediatric Rheumatology, Great Ormond Street Hospital for Children, London, UK; 19 Department of Pediatric Immunology, Rheumatology and Infectious Diseases, University of Amsterdam, Amsterdam, The Netherlands; 20 Emma Children's Hospital, Amsterdam University Medical Centres, Duivendrecht, The Netherlands; 21 Department of Paediatric Rheumatology, University of Cape Town, Rondebosch, Western Cape, South Africa; 22 Department of Paediatric Nephrology, Great Ormond Street Hospital for Children, London, UK

**Keywords:** Lupus Erythematosus, Systemic, Health services research, Glucocorticoids, Autoimmune Diseases, Outcome and Process Assessment, Health Care

## Abstract

**Objectives:**

Application of ‘treat-to-target’ (T2T) in childhood-onset systemic lupus erythematosus (cSLE) may improve care and health outcomes. This initiative aimed to harmonise existing evidence and expert opinion regarding T2T for cSLE.

**Methods:**

An international T2T Task Force was formed of specialists in paediatric rheumatology, paediatric nephrology, adult rheumatology, patient and parent representatives. A steering committee formulated a set of draft overarching principles and points-to-consider, based on evidence from systematic literature review. Two on-line preconsensus meeting Delphi surveys explored healthcare professionals’ views on these provisional overarching principles and points-to-consider. A virtual consensus meeting employed a modified nominal group technique to discuss, modify and vote on each overarching principle/point-to-consider. Agreement of >80% of Task Force members was considered consensus.

**Results:**

The Task Force agreed on four overarching principles and fourteen points-to-consider. It was agreed that both treatment targets and therapeutic strategies should be subject to shared decision making with the patient/caregivers, with full remission the preferred target, and low disease activity acceptable where remission cannot be achieved. Important elements of the points-to-consider included: aiming for prevention of flare and organ damage; glucocorticoid sparing; proactively addressing factors that impact health-related quality of life (fatigue, pain, mental health, educational challenges, medication side effects); and aiming for maintenance of the target over the long-term. An extensive research agenda was also formulated.

**Conclusions:**

These international, consensus agreed overarching principles and points-to-consider for T2T in cSLE lay the foundation for future T2T approaches in cSLE, endorsed by the Paediatric Rheumatology European Society.

## Introduction

Childhood-onset systemic lupus erythematosus (cSLE, also known as Juvenile-onset SLE, JSLE) is a multisystem chronic autoimmune/inflammatory disease. When compared with patients with adult-onset disease, children and adolescents with SLE have higher disease activity and medication burden, more widespread and severe organ manifestations, and higher incidence of renal, cardiovascular and neuropsychiatric involvement than adult-onset SLE (aSLE).^1–4^ Despite 10-year survival having improved,^5^ standardised mortality rates remain higher in cSLE (18.3 in cSLE, 3.1 in aSLE).^6^ cSLE patients have significantly lower health-related quality of life (HRQOL) than their peers^7^ with the majority having accrued permanent organ damage at a young age,^8–10^ suffering high rates of future adult unemployment.^11^

A treat-to-target approach (T2T) is increasingly adopted in chronic diseases, whereby treatment is adjusted or escalated until a specific predefined goal or ‘target’ is achieved, consequently improving disease outcomes. T2T is part of routine clinical care in rheumatoid arthritis, hypertension and diabetes, underpinned by studies demonstrating improvements in long-term outcomes.^12–15^ There is considerable international interest in T2T in cSLE,^16–18^ and aSLE,^19–21^ with the hope that T2T will enable use of existing treatments in a structured way, with the aim of controlling disease activity, preventing organ damage, improving HRQOL, and ultimately improving survival.^21–23^

Internationally agreed principles and recommendations for T2T have laid the foundation for T2T in aSLE,^21^ and Juvenile Idiopathic Arthritis (JIA).^24^ In JIA, clinical trial data already support its use.^25–27^ The TARGET LUPUS research programme: Targeting disease, Agreeing Recommendations and reducing Glucocorticoids through Effective Treatment, in LUPUS’,^16 17^ aims to develop T2T for cSLE. TARGET LUPUS has completed a multicentre qualitative study exploring patient/parental views of T2T^16^ and demonstrated that aSLE-derived low disease activity and remission targets were achievable in UK cSLE patients, associated with significant reductions in flares and new damage.^17^ The TARGET LUPUS programme has a dedicated public and patient involvement (PPI) group (ten cSLE patients, aged 12–27 years) which supports the Task Force, that has also been involved in initiatives to improve communication of the concept of T2T to children and young people,^28^ development of funding applications as co-applicants, and has enabled our group to gain a patient perspective on the studies that have been undertaken as part of the programme to date.^29 30^

Here, an international Task Force was convened to determine if T2T overarching principles and recommendations, summarised here as ‘points-to-consider,’ could be formulated for cSLE, to inform development of future target-based approaches.

## Methods

The methodology was closely aligned with previous T2T initiatives.^21 24^ A cSLE T2T International Task Force was established (July 2021), consisting of two patient representatives, a parent and 20 paediatric specialists, with extensive cSLE clinical and research expertise (14 paediatric rheumatologists, 2 combined paediatric/adult rheumatologists, 4 nephrologists (including collaborators)), an adult rheumatologist with extensive experience of aSLE T2T (EFM), and 2 representatives of the Steering Committee (EMDS, MWB).

Task Force members were invited through the Paediatric Rheumatology European Society (PReS), the Childhood Arthritis and Rheumatology Research Alliance (CARRA), the UK JSLE Study Group, and the UK British Association for Paediatric Nephrology. Self-nominated members were selected according to the following criteria: (A) consultant/specialist in paediatric rheumatology (≥5 years), recognised nationally/international as cSLE expert; (B) treated >10 patients with cSLE over the last 2 years; (C) lead/represent national/international cSLE organisation/group; (D) representative of a subspecialist group(s) that is integral to the care of patients with cSLE (eg, nephrology); or (E) expertise in leading T2T initiatives in SLE or related conditions. The final Task Force committee was chosen based on the applicants’ expertise, balancing representation of the different professional organisations, geographical distribution across all five continents, and availability to participate in all steps of the consensus process.

The Single Hub and Access point for paediatric Rheumatology in Europe (SHARE) initiative published recommendations for the diagnosis and management of cSLE in 2017, based on systematic literature searches performed in 2013,^31–33^ involving some Task Force members (MWB, SSMK, DS-M, TA, CAP, AR). Searches were updated to capture new evidence arising.^34^

Provisional overarching principles and points-to-consider for T2T in cSLE were formulated by the steering committee, considering literature on: (A) targeted therapeutic approaches in cSLE and aSLE; (B) the SHARE recommendations^31–33^; (C) new evidence published since the SHARE literature searches^34^; (D) aSLE T2T recommendations^21^ and (E) JIA T2T recommendations.^24^ These were sent to the Task Force as part of two Delphi surveys (1a/1b). In Delphi 1a, each draft overarching principles and point-to-consider was stated with the evidence underpinning it. Participants were asked if they agreed with its inclusion and given the opportunity to provide comments/suggestions/modifications. Delphi 1a results were shown in Delphi 1b together with revised overarching principles and points-to-consider. Participants were asked if they agreed with the revised overarching principles and points-to-consider and given the opportunity to provide further suggestions.

A virtual consensus meeting (November 2021) was attended by 18 cSLE T2T Task Force members (15 paediatric rheumatologists, 2 dual-trained in rheumatology/nephrology and 1 paediatric nephrologist), with representation from West/East Europe, Africa, Australia, Asia, North and South America. The meeting was chaired by MWB, facilitated by EMDS (both non-voting members). Two patients (NM and LB) and one parent (JA) attended, representing the views of patients/families (non-voting members).

Modified nominal group technique (NGT)^35^ was used to ensure equal participation among Task Force members. The chair (MWB) and facilitator (EMDS) framed each overarching principle and point-to-consider and shared results from Delphi surveys alongside relevant literature/unpublished data from the UK JSLE Cohort Study. Each participant had the opportunity to share opinions for 1 minute without interruption. After all members had commented, participants voted anonymously for their preferred option using an online poll. Agreement of ≥80% of attendees was required when considering consensus as ‘achieved’. When the vote yielded <80% consensus, the overarching principle/point-to-consider was rediscussed and modified, followed by further voting rounds until consensus was achieved (where possible). Each point-to-consider was graded for the level of evidence (LOE, 1–4 scale), and the grade of the point-to-consider (GOR, A to D scale), in accordance with EULAR standardised operating procedures.^36^ The final overarching principles and points-to-consider were reviewed and endorsed by the PReS Executive Council and PReS cSLE Working Party Chair, on behalf of the Society.

## Results

The literature review revealed that no randomised controlled trials had evaluated T2T in cSLE or aSLE. ASLE recommendations for T2T were published in 2014, including four principles and 11 recommendations, supporting targeting of remission, prevention of flares and damage, addressing factors that impact on HRQOL, minimising corticosteroid treatment, recognition of lupus nephritis (LN) and antiphospholipid syndrome (APS) and assessment/treatment of comorbidities.^21^ The SHARE recommendations on the diagnosis and treatment of cSLE,^33^ LN^31^ and APS^32^ were considered during both the Delphi and consensus stages of this project. Where particular SHARE recommendations linked to a cSLE T2T overarching principle or point-to-consider this is detailed below, along with any new evidence since 2013 when the SHARE literature searches were completed (review of new evidence published separately^34^). Broadly, the SHARE recommendations relating to standardised disease activity/damage assessment, follow-up frequency, growth, renal biopsies, treatment of LN, tapering of glucocorticoids, escalation of DMARDS, APS and hydroxychloroquine were all of relevance to the overarching principles and points-to-consider for T2T in cSLE.^31–33^

JIA is the the most common paediatric rheumatic disease, with recent evidence supporting use of T2T approach in polyarticular JIA,^26^ DMARD naïve JIA patients,^25^ and systemic-onset JIA.^27^ These clinical trials were preceded by the development of six principles and eight recommendations for T2T in JIA, agreed by an International Task Force of paediatric rheumatologists. The main treatment target recommended was remission, with minimal/low disease activity a secondary target. Aspects of the principles/recommendations which are of particular relevance to paediatric practice include the need to involve both parents and patients in share decision making and that one of the goals of JIA treatment should be to ‘optimise growth and development’.^37^

### Overarching principles

The Task Force agreed four overarching principles be considered when implementing cSLE T2T points-to-consider (table 1).

**Table 1:** Overarching principles for T2T in cSLE

Overarching principles	Agreement (%)
1. Through shared decision making, both treatment targets and therapeutic strategies for cSLE should be based on individual patients’ disease characteristics and agreed between patients, caregivers and the multidisciplinary healthcare team.	100
2. Treatment of cSLE should aim to ensure long-term survival, prevent organ damage, and optimise health-related quality-of-life. These goals are achieved through control of disease activity, minimising comorbidities and drug toxicity, optimising function, mental well-being, growth, development, education and social participation.	100
3. The management of cSLE requires an understanding of its diverse manifestations, which have to be targeted using patient centred, individualised treatment strategies and a multidisciplinary approach.	100
4. Patients with cSLE require regular and long-term follow-up. This includes monitoring of disease activity, assessment of drug toxicity and adherence, and adjustment of therapy to achieve and maintain their treatment target.	100

Agreement (%) indicates percent of task force members agreeing on the overarching principle during the final voting round of the consensus meeting.^118^

cSLE, childhood-onset systemic lupus erythematosus; T2T, treat-to-target.

#### Overarching principle 1: through shared decision making, both treatment targets and therapeutic strategies for cSLE should be based on individual patients’ disease characteristics and agreed between patients, caregivers and the multidisciplinary healthcare team

In-keeping with SHARE^33^ and JIA T2T principles,^24^ the Task Force emphasised that patients/caregivers should play an important role in setting the treatment target and therapeutic strategy, based on patient characteristics (eg, prior treatment, disease activity, existing damage, prior treatment adherence, characteristics associated with poor outcome). Recognising the heterogeneity of cSLE, the Task Force underlined the importance of decisions also being agreed by the ‘multidisciplinary healthcare team’. The Task Force recognised that shared decision making may be more challenging to implement in paediatrics due to potential power imbalance between children, adolescents and caregivers.^38^ Certain types of disease damage (eg, end-stage renal failure) may result in an inability to reach common targets, in which case an individualised target should be agreed through shared decision making.

#### Overarching principle 2: treatment of cSLE should aim to ensure long-term survival, prevent organ damage and optimise HRQOL

These goals are achieved through control of disease activity, minimising comorbidities and drug toxicity, optimising function, mental well-being, growth, development, education and social participation.

Overarching principle 2 includes two statements emphasising that T2T should aim to improve long-term outcomes (mortality, damage and HRQOL) and that a multifaceted approach is required; controlling disease activity, managing comorbidities, drug toxicity, improving function, mental well-being, growth, development, education, and social participation. cSLE morbidity relates in part to treatment, in-particular high dose or chronic use of glucocorticoids which although aimed at reducing the risk of disease flare, carries the associated risk of long-term steroid-induced damage.^39^ The importance of education, social participation and subsequent employment were emphasised by patient/parent representatives. ‘Growth and development’ reflect health status but also impact on self-perception of ‘being normal,’^16^ warranting a specific mention. A team approach, involving medical subspecialists (eg, nephrology, rheumatology, neurology), primary care practitioners, psychologists, specialist nurses, physiotherapists, occupational therapists, social workers and education teams is therefore warranted, to optimise patient outcomes.

#### Overarching principle 3: the management of cSLE requires an understanding of its diverse manifestations, which have to be targeted using patient centred, individualised treatment strategies and a multidisciplinary approach

This overarching principle acknowledged the different clinical phenotypes of cSLE, which must be recognised if the optimal target and treatment strategies are to be selected for an individual patient. It also underlines the need for collaboration between different medical specialists, allied healthcare professionals and nurses to support children and adolescents to reach their targets.

#### Overarching principle 4: patients with cSLE require regular and long-term follow-up. This includes monitoring of disease activity, assessment of drug toxicity and adherence, and adjustment of therapy to achieve and maintain their treatment target

Maintenance of the target and ‘tight control’ underpin the T2T approach.^21^ When the target is lost, it is important to assess if this has been precipitated by a flare of the disease, treatment intolerance or non-adherence.^31 40^

### Points-to-consider

The Task Force agreed on 14 points-to-consider, the details of which are presented in table 2.

**Table 2:** Points-to-consider for T2T in cSLE

Points-to-consider	Agreement (%)	LOE	GOR
1. The treatment target of cSLE should be disease remission.	87	3	C
2. In patients where remission cannot be achieved, low disease activity is an alternative target.	100	3	C
3. Prevention of flares should be targeted, as an important therapeutic goal	100	2	B
4. Patients with clinically inactive disease and persistent low complement and/or elevated anti-ds-DNA antibody titres require close monitoring. Therapy should not be escalated solely on these results.	100	3	C
5. Prevention of organ damage accrual, measured using a validated SLE damage index, should be a major therapeutic goal in cSLE.	100	2	B
6. Factors influencing HRQOL, such as fatigue, pain, mental health, educational challenges, and medication side effects should be proactively addressed, through a multidisciplinary approach.	94	3	C
7. Early recognition and treatment of renal involvement is strongly recommended.	100	3	C
8. In patients with histologically proven class III, IV and/or V lupus nephritis, following induction therapy, a period of immunomodulatory maintenance therapy lasting at least 3 years is recommended.	94	3	D
9. Maintenance treatment should aim for the lowest glucocorticoid dosage needed to control disease, through optimisation of immunomodulatory therapy.	100	2	C
10.Prevention and treatment of antiphospholipid antibody related morbidity should be a long-term therapeutic goal.	100	3	C
11.All patients should be prescribed hydroxychloroquine routinely, unless there are contraindications.	94	2	B
12.Prevention and control of comorbidities should be a treatment target.	94	4	D
13.Frequent assessment is recommended to ensure the patient is on the correct trajectory to achieve their target, using standardised assessment tools.	100	4	D
14.Once the target has been achieved, it should be sustained. Ongoing monitoring should occur to ensure maintenance of the target.	100	3	C

During the discussion of points-to-consider 1–5 and 14, one or two task force participants were absent from the on-line meeting due to urgent commitments. Each point-to-consider was graded for the LOE on a scale of 1–4, and the GOR on a scale from A (highest) to D (lowest), in accordance with EULAR standardised operating procedures for EULAR-endorsed recommendations.^26^ LOE 1A: From meta-analysis of randomised controlled trials, 1B: From at least one randomised controlled trial, 2A: From at least one controlled study without randomisation, 2B: From at least one type of quasi-experimental study, 3: From descriptive studies, such as comparative studies, correlation studies or case–control studies, 4: From expert committee reports or opinions and/or clinical experience of respected authorities. GOR A: directly based on category I evidence, B: directly based on category II evidence or extrapolated points-to-consider from category I evidence, C: directly based on category III evidence or extrapolated points-to-consider from category I or II evidence, D: directly based on category IV evidence or extrapolated point-to-consider from category II or III evidence. Agreement (%) indicates percent of experts agreeing on the point-to-consider during the final voting round of the consensus meeting.^27^

cSLE, childhood-onset systemic lupus erythematosus; GOR, grade of the ensuing point-to-consider; HRQOL, health-related quality of life; LOE, level of evidence; T2T, treat-to-target.

#### Point-to-consider 1: the treatment target of cSLE should be disease remission

Remission is proposed as the ultimate treatment target. However, defining cSLE-specific remission was beyond the scope of the current initiative. To inform development of paediatric target definitions, 93% of the Task Force agreed that overall targets are most appropriate (rather than ‘organ-specific’ targets), recognising that it is unusual for one organ to be involved and treated in isolation in cSLE.

Two previous studies investigated the achievability of aSLE-defined clinical remission (CR) on/off treatment in cSLE, using definitions from the 2017 Definition of Remission in SLE Framework.^41^ The UK JSLE Cohort Study (n=430, 4738 visits, median follow-up 2 years) showed CR on/off-treatment to be achieved by 61% and 31% of patients, respectively.^17^ Similarly, a Dutch cSLE Cohort (n=51, 700 visits, median follow-up 3 years), demonstrated CR on/off-treatment to be achieved by 53%/22% of patients, respectively.^18^

In UK patients, achievement of CR on-treatment was associated with significantly reduced hazards of severe flare (HRs 0.19, 95% CI 0.15 to 0.24) and new damage (HR 0.27, 95% CI 0.14,0.50, both p<0.001). Achievement of CR off-treatment was associated with further reduction in the hazards of severe flare (HR 0.13 (95% CI 0.09 to 0.20), 95% CI 0.15,0.24) and new damage (HR 0.10, 95% CI 0.07 to 0.16, both p<0.001). Statistically, there was no significant difference between the hazards of severe flare when the aSLE definition of LLDAS^19^ or either definition of CR (on/off treatment) were compared (all p_c_>0.05).^17^ The Task Force noted that future work specifically defining paediatric targets should aspire to establishing clear separation between low disease activity and remission.

#### Point-to-consider 2: in patients where remission cannot be achieved, low disease activity is an alternative target

While remission is the ultimate target, this often is unattainable for patients; particularly those with long-standing disease, an aggressive disease course, pre-existing damage, or where drug toxicity is encountered. In such patients, shared decision making was viewed as key to deciding whether low disease activity would be an appropriate initial target. The patient representatives agreed that low disease activity should be recognised as an alternative target, as having only the target of remission could lead to a sense of failure for some patients.

In the UK and the Netherlands, a higher proportion of cSLE patients reached the aSLE LLDAS definition^19^ of low disease activity than CR; 67% of the UK Cohort and 100% of the Dutch cohort.^17 18^ Within the UK Cohort, a greater cumulative duration of time in LLDAS or CR (from 10% to 80% of follow-up), was associated with reduced hazards of severe flare (eg, from 0.68 to 0.05 for LLDAS), supporting pursuit of sustained target attainment.^17^

#### Point-to-consider 3: prevention of flares should be targeted, as an important therapeutic goal

cSLE flares often result in the addition of a short course of high-dose glucocorticoid treatment and/or initiation or intensification of immunosuppressives. Optimisation of immunosuppressant treatment should be systematically pursued, through adequate weight-based drug dosing, assessment of adherence, quantification of drug levels (where possible) to mitigate the difficult balance between treating with glucocorticoids to reduce disease flare versus the risk of steroid-induced damage.^42^ In cSLE, the mean annual flare frequency is associated with shorter time to damage development (HR 2.38/unit increase in flare frequency, p=0.018),^43^ supporting the importance of flare prevention. Cumulative disease activity (reflecting persistent disease activity and flares) in cSLE is also associated with damage,^44^ and development of damage is in turn associated with mortality.^45 46^ With standardised mortality rates significantly higher in cSLE than aSLE,^6^ the importance of preventing flares, damage and consequently mortality are underlined.^46^

#### Point-to-consider 4: patients with clinically inactive disease and persistent low complement and/or elevated anti-ds-DNA antibody titres require close monitoring. Therapy should not be escalated solely on these results

No cSLE studies have specifically looked at the risk of flare in patients with clinically inactive disease and persistent (or on-going) low complement and/or elevated anti-ds-DNA antibody titres. In cSLE, patients have a higher rate of both anti-dsDNA positivity and low C3 levels than in aSLE,^6 47^ particularly in adolescents at diagnosis (vs prepubertal and peripubertal patients).^48^ Low C3 and high anti-ds-DNA antibody titres are associated with early onset LN.^49^ Low C3 levels are also associated with LN at disease onset, and a predictor of subsequent LN development.^50^ The aSLE T2T recommendations use the terms ‘serological activity’,^21^ however, the cSLE Task Force considered this to be ambiguous and preferred a more explicit definition; ‘persistent low complement and/or elevated anti-ds-DNA antibody titres’. Although the Task Force agreed that treatment should not be escalated based solely on such results, considerable effort should be made to rule out any disease activity, with ongoing close monitoring.

#### Point-to-consider 5: prevention of organ damage accrual, measured using a validated SLE damage index, should be a major therapeutic goal in cSLE

Damage in cSLE^8 39 43 44 51–58^ is associated with the cumulative duration of disease activity, disease flares, glucocorticoid and cyclophosphamide treatment.^44 53 55^ Hydroxychloroquine use has been associated with less damage accrual.^43 54^ cSLE related damage has been shown to occur within the first year of disease,^8^ with the vast majority of cSLE patients having developed damage by their early twenties.^9^ Patients are also more likely to report glucocorticoid-related damage (OR 1.7, 95% CI 1.1 to 2.8),^39^ particularly where moderate dose glucocorticoid treatment is used for a prolonged period. Minority race/ethnicity has been associated with increased damage accrual during follow-up in cSLE, therefore, more careful follow-up is required.^59^ In aSLE, early accrual of damage is strongly associated with subsequent damage accrual^60 61^ and mortality.^61 62^ Studies in cSLE have similarly shown damage to be associated with increased mortality risk.^46^ This highlights the need to to limit the duration of active disease/flares. In keeping with SHARE recommendations,^33^ the Task Force therefore agreed that serial measurement of damage alongside T2T approaches is necessary.

#### Point-to-consider 6: factors influencing HRQOL, such as fatigue, pain, mental health, educational challenges and medication side effects should be proactively addressed, through a multidisciplinary approach

In cSLE, HRQOL correlates with fatigue, pain, anxiety and depressive symptoms, with disease activity scores being a poor predictor of impaired HRQOL.^63^ The prevalence of depression symptoms has been shown be up to 59% in cSLE studies, with anxiety affecting up to 37% of patients.^64^ Suicidal ideation is also significantly higher in cSLE patients (14%) compared with healthy controls (4%). Despite this, low rates of access to mental health treatment are described,^65^ highlighting the importance of screening for mental health symptomatology and proactively promoting a multidisciplinary response. cSLE also negatively impacts on school attendance and performance,^66^ while ability to attend school is a key contributor to HRQOL, associated with social, academic and employment benefits.^11 67^ Medication side effects, particularly those affecting physical appearance are also strongly associated with poor HRQOL.^9^ There was high levels of agreement that factors known to impact on HRQOL should be directly addressed in addition to pursuit of disease activity based targets, with support of a multidisciplinary team. This point-to-consider is also in-keeping with aSLE T2T recommendations.^21^

#### Point-to-consider 7: early recognition and treatment of renal involvement is strongly recommended

LN is a major cause of morbidity in cSLE, a common form of damage,^9 68^ and is associated with mortality in cSLE and aSLE.^69–73^ A total of 50%–80% of cSLE patients develop LN^58 74 75^ compared with 40%–50% in aSLE.^1 76^ Delay in renal biopsy (and therefore LN diagnosis) in aSLE is associated with development of end-stage renal disease.^77–79^ In keeping with SHARE recommendations,^31^ the Task Force advocated for vigilance regarding LN recognition and having a low threshold for kidney biopsy.

#### Point-to-consider 8: in patients with histologically proven class III, IV and/or V LN, following induction therapy, a period of immunomodulatory maintenance therapy lasting at least 3 years is recommended

No RCTs have compared different durations of maintenance LN immunomodulatory therapy, and observational data are limited.^80^ In aSLE patients achieving LN CR for at least 12 months; 21/73 (28.7%) flared during tapering of treatment; 38% flared within 3 years of treatment withdrawal; and the remaining 33% did not flare after a median of 102 months follow-up, as per a retrospective study. The risk of flare was lowest for those with the longest duration of stable remission prior to treatment discontinuation.^81^ In RCT open-label extension and observational studies, most LN flares have been demonstrated within 5–6 years of LN onset.^82–86^ SHARE LN recommendations state ‘Although specific paediatric data are missing, maintenance treatment for class III and IV LN should be administered for at least 3 years’.^31^

The Task Force debated extensively whether this point-to-consider should only apply to class III/IV LN or class III, IV and/or V. Class V LN has been included as it is frequently concomitant with class III/IV LN, histological transition from class V to class III/IV LN has been observed,^80^ and recent clinical trials assessing novel immunomodulators have grouped class III/IV/V together.^87^ Future paediatric studies assessing the duration of immunomodulatory maintenance therapy are necessary.

#### Point-to-consider 9: maintenance treatment should aim for the lowest glucocorticoid dosage needed to control disease, through optimisation of immunomodulatory therapy

The Task Force advised that glucocorticoid withdrawal should be gradual, balanced against disease severity and organ involvement. A recent aSLE study comparing maintenance low-dose prednisolone (5 mg/day) versus complete withdrawal, in patients with clinically quiescent disease, showed low-dose maintenance prednisone to be superior in terms of time to first flare (HR 0.2; 95% CI 0.1 to 0.6, p=0.002), occurrence of mild/moderate flares (RR 0.2 (95% CI 0.1 to 0.8), p=0.012) and moderate/severe flares (RR 0.1 (95% CI 0.1 to 0.9), p=0.013).^88^ However, there is a dose-related association between glucocorticoid exposure and damage accrual.^89^ cSLE patients are at increased risk of glucocorticoid-related damage when compared with aSLE.^39^ In aSLE patients with no clinical or serological disease activity, accrual of damage is independently associated with time-adjusted mean prednisolone dose (HR 1.14, 95% CI 1.03 to 1.26, p=0.0117), emphasising that there is no ‘safe low-dose’ of glucocorticoid.^90^ In addition, in patients with cSLE, high cumulative doses of glucocorticoid (>400 mg/kg) adversely impact on growth and pubertal development.^91^

Based on currently available data it is not possible to define a ‘safe low dose’ of maintenance glucocorticoid for cSLE, and further studies are required to investigate this issue (see research agenda, box 1). In-keeping with the SHARE recommendations,^33^ the Task Force agreed that ‘optimisation of immunomodulatory therapy’ should be emphasised as means to achieving the ‘lowest glucocorticoid dosage’.

Box 1 **Research agenda relating to the fourteen childhood-onset systemic lupus erythematosus (cSLE) treat-to-target (T2T) points-to-consider**

**Point-to-consider 1**

- Development of paediatric specific definition(s) of remission which maintains sufficient homology with adult-onset SLE (aSLE) remission target definitions to promote life-course collaborative studies between the paediatric and adult rheumatology communities.
- Validation of paediatric specific definition(s) of remission in international cohorts and prospective studies.
- Prospective randomised clinical trial comparing targeted versus standard care in cSLE.
- Longitudinal clinical studies investigating the longer-term outcomes of targeted treatment in cSLE.
- Defining the most effective means of ensuring meaningful shared decision making within routine clinical care environment.

**Point-to-consider 2**

- Development of a paediatric specific definition of Low Disease Activity which maintains sufficient homology with aSLE remission target definitions to promote life-course collaborative studies between the paediatric and adult rheumatology communities.
- Compare outcomes associated with attainment of paediatric specific definitions of remission and low-disease activity in cSLE.

**Point-to-consider 3**

- Further validation of flare definitions and flare assessment tools.
- Prospective studies to investigate early intensive therapy and the principle of induction/maintenance in non-renal lupus, as a means of preventing flares.

**Point-to-consider 4**

- Undertake analyses within existing longitudinal cohorts and prospective clinical studies, looking at risk of flare in patients with clinically quiescent disease and persistent low complement and/or elevated anti-ds-DNA antibody titres.

**Point-to-consider 5**

- Undertake further analyses within existing longitudinal cohorts and prospective clinical studies, assessing early accrual of damage as a predictor for subsequent damage accrual and mortality (as in aSLE).
- A study specifically assessing whether damage prevention leads to improvements in HRQOL.
- Prospective studies to investigate early intensive therapy and the principle of induction/maintenance in non-renal lupus, as a means of preventing damage accrual.

**Point-to-consider 6**

- Further studies of non-inflammatory factors influencing health related quality of life in cSLE.
- Development and validation of a set of key patient-reported outcomes that capture factors influencing health related quality of life, including fatigue, pain, mental health, educational challenges, and medication side effects.
- Development of multidisciplinary interventions that could be used in parallel to a T2T approach in cSLE, to address parallel patient targets, eg. fatigue, pain, mental health, educational challenges and medication side effects.

**Point-to-consider 7**

- Further assessment of haematuria and/or leukocyturia as a prompt for consideration of renal biopsy (as compared tocompared with persistent mild proteinuria and/or impaired glomerular filtration rate).
- Undertake analyses within existing longitudinal cohort to assess if delay in renal biopsy (and therefore diagnosis of Lupus Nephritis) is associated with development of end-stage renal disease in cSLE.

**Point-to-consider 8**

- Prospective clinical studies comparing different durations of maintenance immunomodulatory therapy for different lupus nephritis classes.
- Undertake analyses within existing longitudinal cohort and prospective studies to assess rates of histological transition from class V to class III/IV lupus nephritis in cSLE patients undergoing a repeat renal biopsy (performed either per protocol or during an lupus nephritis flare).

**Point-to-consider 9**

- Undertake analyses within existing longitudinal cohorts and prospective clinical studies to assess for a dose-related association between glucocorticoid exposure and damage accrual, and whether a ‘safe’ lower dose of glucocorticoid can be identified.
- Prospective clinical studies comparing high-dose versus low-dose intravenous methylprednisolone treatment regimens in terms of efficacy, side effects and damage accrual.
- Studies on glucocorticoid withdrawal, comparing flare rates in cSLE patients with clinically quiescent disease on maintenance low-dose prednisolone versus patients where glucocorticoids have been withdrawn.

**Point-to-consider 10**

- Studies to better define which patients would benefit from treatment aimed at primary prevention of thrombosis.
- Therapeutic studies in cSLE antiphospholipid syndrome with different immunosuppressives/immunomodulators.
- Therapeutic studies in cSLE antiphospholipid syndrome to provide evidence for different anticoagulation and anti-aggregation regimens.
- Studies assessing the feasibility of discontinuing anticoagulant therapy following immunomodulation, where the production of antiphospholipid antibodies has been suppressed.

**Point-to-consider 11**

- Further studies to assess if hydroxychloroquine must be recommended or not in every patient with cSLE.
- Further studies investigating the assessment of serum hydroxychloroquine drug level monitoring.
- Studies assessing long-term toxicity/side effect associated with hydroxychloroquine in cSLE.

**Point-to-consider 12**

- Development and validation of a structured assessment to longitudinally assess for comorbidities in cSLE, for used within routine clinical practice.
- Develop standardised interventional approaches to address comorbidities once they have been identified.

**Point-to-consider 13**

- Determine the optimal frequency of target assessment and treatment escalation.

**Point-to-consider 14**

- Prospective clinical studies assessing the impact of varying percentages of follow-up time in target (low disease activity and remission), and how this impacts on flare rates and new damage accrual.
- Studies assessing approach to withdrawal of therapy when remission has been achieved.

#### Point-to-consider 10: prevention and treatment of antiphospholipid antibody related morbidity should be a long-term therapeutic goal

APS is rare in cSLE. In 2004, the international Ped-APS registry identified 121 cases across a multinational study, 41% in association with cSLE or ‘lupus-like’ disease.^92 93^ A study involving 27 Brazilian centres identified cSLE related APS in 67/1519 (4%) patients; 40/67 (60%) developed venous thrombosis; 35/67 (52%) arterial thrombosis; 9/67 (13%) small vessels thrombosis and 3/67 (4%) mixed venous and arterial thromboses. No patients had catastrophic APS.^94^ A Dutch cSLE long-term follow-up study identified antiphospholipid antibody positivity as an independent predictor associated with damage accrual (OR 3.56, p=0.026).^9^ Antiphospholipid antibody positivity is also associated with mortality in cSLE.^95^

The Task Force discussed this point-to-consider extensively, particularly related to prevention of APS related morbidity. They noted the detailed SHARE recommendations for APS, and supported screening for antiphospholipid antibodies (lupus anticoagulant, anticardiolipin IgG and IgM and anti-β2-glycoprotein-I IgG and IgM) in all cSLE patients in accordance with the SHARE recommendations.^32^ Specifying a particular treatment approach for APS was deemed beyond the scope of these points-to-consider.

#### Point-to-consider 11: all patients should be prescribed hydroxychloroquine routinely, unless there are contraindications

Antimalarial treatment is considered a quality indicator in North America,^59^ with a large retrospective cSLE cohort (n=473) demonstrating antimalarial treatment to be associated with protection against new damage accrual.^54^ Hydroxychloroquine blood levels are inversely correlated with disease activity in cSLE,^96^ with low concentrations predictive of flare.^97^ The Task Force noted hydroxychloroquine toxicity to be rare in cSLE.^98^ In aSLE, total daily hydroxychloroquine doses of >5 mg/kg, renal impairment and certain concomitant medications (eg, Tamoxifen) are associated with hydroxychloroquine retinopathy risk.^99^

The aSLE T2T recommendations^21^ state that ‘serious consideration should be given to the use of antimalarials’, but in-keeping with the SHARE recommendations,^33^ the Task Force was more explicit that hydroxychloroquine ‘should be prescribed routinely’, unless contraindicated. The point-to-consider uses the term ‘hydroxychloroquine’ as current evidence largely relates to this antimalarial compound.^100^

#### Point-to-consider 12: prevention and control of comorbidities should be a treatment target

The Task Force recognised the importance of prevention, assessment and control of cSLE-associated comorbidities including hypertension, hypercholesterolaemia, insulin resistance/diabetes, antiphospholipid antibody related morbidity, bone health/osteopenia/osteoporosis, prevention of infection through adequate vaccination, assessment and provision of support for mental health, delivered through a multidisciplinary team where possible. The high prevalence of depression, anxiety,^64^ and suicidal ideation in cSLE,^65^ highlights the importance of screening for comorbidity related to mental health in particular.

Of particular importance, many disease-related factors increase cardiovascular disease risk in cSLE (eg, chronic inflammation, endothelial dysfunction, antiphospholipid antibodies) adding to treatment related issues (eg, glucocorticoid and cyclophosphamide associated metabolic changes), and lifestyle/traditional factors (deconditioning, hypercholesterolaemia/poor diet/obesity, insulin resistance/diabetes, hypertension).^101^ Younger patients with SLE (18–44 years) have a higher relative prevalence of myocardial infarction (MI, adjusted proportionate morbidity ratio, PMR 1.82 (95% CI 1.03 to 3.26)) compared with both the general population, and older SLE patients (SLE patients aged 45–64 years PMR 1.02 (0.77–1.34); ≥65 years 0.71 (0.54–0.94)), in analyses adjusting for the presence of hypertension, diabetes, chronic renal failure, age, race, hospital characteristics and insurance status.^102^ Optimising disease control in parallel to addressing cardiovascular risk factors is therefore crucial,^103^ to reduce the considerable risk of MI seen in young adults with cSLE.

#### Point-to-consider 13: frequent assessment is recommended to ensure the patient is on the correct trajectory to achieve their target, using standardised assessment tools

The Task Force noted the frequency of assessments in cSLE should be guided by organ manifestations, level of disease activity, disease duration, stage of treatment and any patient characteristics associated with potentially poor outcome. The Task Force stressed that dependent on these factors, weekly, monthly, up to 3-monthly assessment by a cSLE specialist centre may be indicated, in keeping with SHARE.^33^ The Task Force underlined the importance of assessing the trajectory of progress towards target achievement, paying close attention to disease activity (using a standardised tool) and glucocorticoid dosage. It was noted that the frequency of appointments should not mean that particular immunomodulatory therapies are deemed ineffective and discontinued prematurely. Optimisation of immunomodulatory treatments (considering maximal weight based dosage, drug levels and adherence) should be considered prior to switching treatments. Access to care can pose challenges for families and clinicians, and innovative approaches including use of telemedicine^104 105^ and undertaking laboratory investigations locally, may be needed for some visits.

#### Point-to-consider 14: once the target has been achieved, it should be sustained. Ongoing monitoring should occur to ensure maintenance of the target

Evidence supporting this point-to-consider comes from the UK JSLE Cohort Study,^17^ where increased cumulative percentage of time in target (LLDAS or CR on/off treatment) was associated with progressive reduction in the hazard of severe flare, highlighting the importance of sustained target attainment,^17^ in keeping with JIA T2T recommendations.^24^ The Task Force noted that, for many patients, the initial target may be low disease activity, and that once this had been attained it should either be sustained, or a more stringent target considered. Maintenance of the treatment target does not necessarily imply that treatment must remain static and cannot be weaned/stopped or gradually tapered, particularly with regards to glucocorticoid treatment (see point-to-consider 9).

### Research agenda

The Task Force discussions and structured literature review highlighted important issues that remain elusive in relation to evidence informing a future T2T based approach for cSLE. The Task Force therefore developed a research agenda, shown in box 1.

## Discussion

The International cSLE T2T Task Force has developed overarching principles and points-to-consider, which have been endorsed by PReS, representing the first step towards developing a T2T approach for cSLE. These are based on published evidence available to date, derived after two rounds of Delphi surveys and extensive discussions using a modified NGT approach, with excellent agreement between a broad range of international experts. The overarching principles and points-to-consider are intended for experienced clinicians aimed at improving patient care, and are not intended to replace clinical judgement, knowledge, and experience. Patient preferences, shared decision making and equity (or otherwise) of access to care and resources should be taken into consideration at the individual patient level. Development of full principles and recommendations for T2T in cSLE is anticipated, following investigation of the research agenda items detailed above.

The three essential elements of T2T are: (1) a target; (2) a means of measuring whether the target has been achieved and (3) having appropriate interventions to achieve the target. There are many ways to treat cSLE, and T2T does not recommend specific treatments to achieve the goals outlined. In cSLE, recent studies involving UK and Dutch cSLE cohorts have demonstrated that aSLE-derived targets (LLDAS/CR) are attainable in cSLE,^17 18^ and associated with reduced risk of both severe flare and new damage.^17^ However, future initiatives are needed to derive and validate cSLE-specific targets, ensuring that they are applicable to cSLE while maintaining sufficient unity to facilitate future T2T studies including adolescents and adults together are possible. An obvious example of how the existing aSLE definitions may be inappropriate for cSLE relates to the glucocorticoid related criteria which do not currently include a weight-based cut-off for glucocorticoid dosing. Use of the existing aSLE-derived targets could therefore allow treatment with a relatively high dose of glucocorticoid for younger children with cSLE. To determine whether existing targets have been achieved requires regular monitoring of validated disease activity measure (eg, SLEDAI or BILAG), a record of the physicians global score, and immunomodulatory treatment and glucocorticoid dosage.^19 20 41^ This may be aided by an app-supported method for target calculation.^106^

Therapeutic options for cSLE remain limited, with only one biologic (belimumab)^107^ approved for use in cSLE, and Rituximab used ‘off-label’ for refractory cases.^108–112^ A T2T strategy is therefore attractive and timely, offering an opportunity to use existing treatments in a structured/targeted way with the anticipated aim of controlling disease activity at an earlier stage in order to prevent damage and improve longer term outcomes. Emerging evidence from cohort studies suggest that early introduction of immunosuppressive treatment with mycophenolate mofetil was associated with increased attainment of LLDAS.^18^ New therapeutic agents are likely to further strengthen the T2T approach. The Task Force acknowledged variable accessibility to medications may lead to disparities in the proportion of patients who are able to attain targets across countries or regions.

Among the outstanding research priorities (box 1), two are deemed urgent: (1) developing paediatric specific target definition(s) and (2) establishing if there is a dose-related association between glucocorticoid exposure and damage accrual in cSLE. For disease flares, higher doses of intravenous pulse methylprednisolone are recommended in cSLE as compared with aSLE,^31 33 113–115^ with comparisons of high-dose versus low-dose intravenous methylprednisolone regimens in terms of efficacy, side effects and damage accrual lacking. For patients in remission on low dose glucocorticoids, a key priority is to assess whether a ‘relatively safe’ low dose of glucocorticoid can be identified. This is particularly important as cSLE patients are at high risk of glucocorticoid related damage.^39^

The importance of including patients’/caregivers’ views in the development of these T2T overarching principles and points-to-consider was crucial and pivotal in many of the Task Force discussions.^16 116^ The TARGET LUPUS PPI group has supported this process. Two patients and a parent attended the consensus meeting to represent the views of patients/carers. This was extremely important, helping cSLE experts resolve areas of disagreement and ensuring the needs of patients were addressed, particularly when considering potential treatment side effects and quality of life. Our patient representatives highlighted that a T2T approach could pose socioeconomic challenges for families, potentially exacerbating known health disparities in cSLE,^117^ underlining the need for provision of socioeconomic support when testing a T2T approach. The PPI group will continue to inform all cSLE Task Force initiatives.

## Conclusions

T2T overarching principles and points-to-consider for cSLE have been developed by an international Task Force, including paediatric rheumatologists, nephrologists, adult rheumatologists and patients/caregivers, endorsed by PReS. Building on published evidence available to date, excellent levels of agreement were achieved. These overarching principles and points-to-consider form a key initial step towards developing a T2T approach and establishing its role within cSLE patient care.
